# Successful Application of Extra‐Intracranial Microanastomosis in the Management of Giant Intracranial Aneurysms: A Clinical Case

**DOI:** 10.1002/ccr3.73550

**Published:** 2026-09-15

**Authors:** Ruzikulov M. Mazhidovich, Kashif Qureshi, Kariev G. Maratovich, Rasulov S. Orzukulovich, Matmusaev M. Makhmudovich, Murtaja Satea, Khazratkulov R. Bafoevich, Matmusaev M. Makhsudovich, Ismail Bozkurt, Kivanc Yangi, Bipin Chaurasia

**Affiliations:** ^1^ Department of Neurosurgery Republican Specialized Scientific and Practical Medical Center of Neurosurgery Tashkent Uzbekistan; ^2^ Department of Neurosurgery Yale School of Medicine New Haven Connecticut USA; ^3^ Department of Neurosurgery College of Medicine, University of Warith Al‐Anbiyaa Holy Karbala Iraq; ^4^ Department of Neurosurgery Medical Park Ankara Hospital Ankara Türkiye; ^5^ Department of Neurosurgery Barrow Neurological Institute Phoenix Arizona USA; ^6^ Department of Neurosurgery Neurosurgery Clinic Birgunj Nepal

**Keywords:** cerebral aneurysms, cerebral revascularization, coil embolization, partially thrombosed aneurysm

## Abstract

We report a case of a giant middle cerebral artery aneurysm treated with a staged strategy: initial extracranial–intracranial revascularization via superficial temporal artery–M4 bypass, followed by definitive endovascular aneurysm exclusion with detachable microcoil embolization. This technique allowed preservation of cerebral blood flow and successful treatment of the aneurysm.

## Key Clinical Message

Cerebral aneurysms may pose a high risk of rupture. Revascularization surgery is a viable option to manage complex aneurysm cases. In selected patients, revascularization can be combined with endovascular techniques to optimize aneurysm control.

## 1 Introduction

Despite the high prevalence of intracranial aneurysms in the general population (3%) [1, 2] and the devastating consequences of their rupture [3], the pathological mechanisms underlying their formation still remain poorly understood. This pathology is associated with high mortality in ruptured intracranial aneurysms with subarachnoid hemorrhage (SAH), with a mortality rate of approximately 27%–44% [4]. Established risk factors for the development of intracranial aneurysms include smoking, alcohol consumption, arterial hypertension, atherosclerosis, female sex, older age, oral contraceptive use, Type III collagen deficiency, anatomical variations of the circle of Willis, brain arteriovenous malformations, viral infection, pituitary tumors, and various human leukocyte antigen (HLA)‐related factors [5, 6, 7, 8, 9, 10, 11, 12]. The incidence is generally higher in women. However, in young adults, it may be more frequently seen in men [13].

Smoking is considered one of the major risk factors for rupture of intracranial aneurysms and has been associated with an imbalance between elastase and α1‐antitrypsin, as well as a transient increase in blood pressure. Although smokers may generally have lower baseline blood pressure than nonsmokers, smoking can cause blood pressure to rise for several hours. This short‐term increase may contribute to aneurysm rupture. Alcohol consumption has also been associated with an increased risk of rupture, possibly due to its vasoactive effects on the cerebral circulation.

Another important factor is aneurysm morphology and complexity. Complex aneurysms include those with a wide neck, calcified neck or dome, functionally important branches arising from the dome, multilocular aneurysms, partially thrombosed aneurysms, aneurysms that are difficult to localize, and giant aneurysms [14]. Currently, there are several treatment options. The first is microsurgical clipping of the aneurysm neck, trepanation of the aneurysm, and proximal ligation [14, 15]. The second is endovascular therapy, including stenting of the parent artery with or without flow‐direction control, occlusion of the aneurysmal cavity with a balloon or detachable microcoils, and embolization [16]. However, for complex aneurysms, these methods do not always provide the desired surgical result of isolating the aneurysm from the bloodstream while maintaining patency of the parent artery.

In recent years, revascularization techniques have increasingly been used for complex aneurysms. These techniques include creating intracranial or extracranial bypasses to preserve cerebral blood flow in the aneurysm‐affected vascular territory, using either high‐flow or low‐flow grafts as required [17, 18, 19]. The introduction of cerebral revascularization techniques has significantly improved the treatment of complex aneurysms by enabling aneurysm exclusion while maintaining blood flow to the distal vascular territory, thereby reducing the risk of ischemic neurological deficits in selected patients [17, 18].

In this article, we report a successful case of microsurgical treatment of a complex MCA aneurysm using two‐stage revascularization.

## 2 Case History/Examination

A 51‐year‐old male patient was admitted with a 2‐week history of severe headache and an episode of loss of consciousness, without an identifiable precipitating factor. He was initially treated at a local hospital and stabilized. On presentation, there was no clinical or radiological evidence of SAH, and neurological examination revealed no focal deficit. The clinical course and imaging findings were therefore consistent with a symptomatic, partially thrombosed giant aneurysm rather than acute aneurysmal rupture.

## 3 Methods (Differential Diagnosis, Investigations, and Treatment)

**FIGURE 1:**
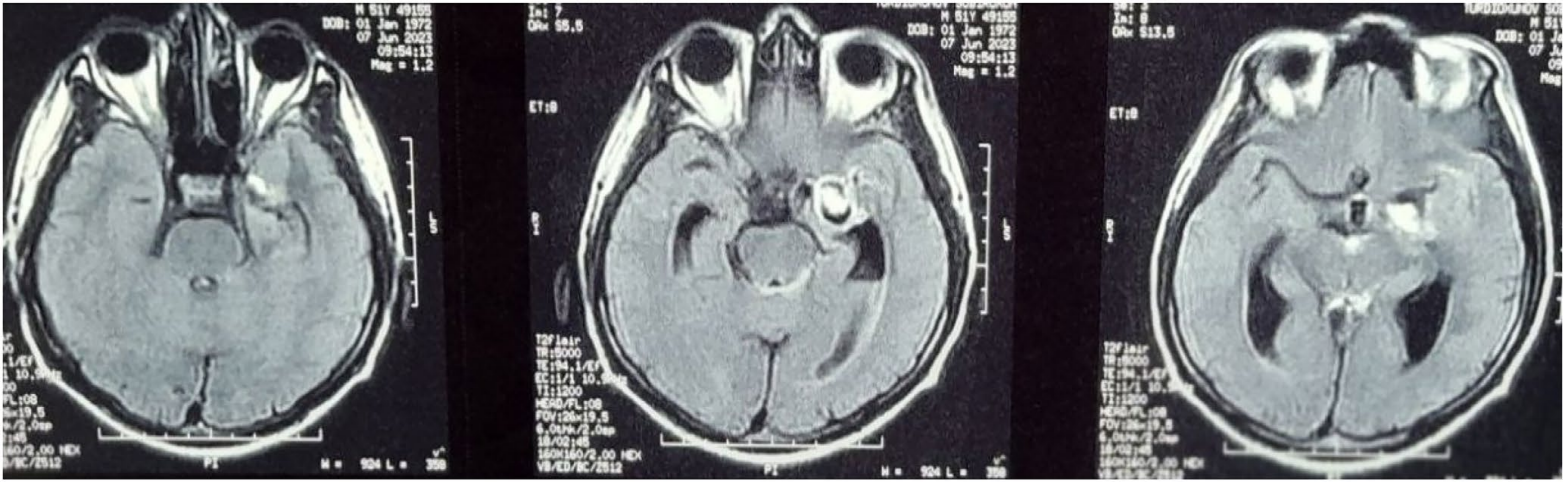
Brain MRI demonstrating a giant left MCA aneurysm. The aneurysm shows variable signal intensity, including T1 hyperintensity (first image), T2 hypointensity (middle), and heterogeneous signal on T2‐FLAIR (last), suggesting thrombosis of most of the aneurysm sac. The peripheral T1 hyperintensity corresponds to laminated mural thrombus of differing ages, whereas the small residual patent lumen appears as a central flow void. The T2 hypointensity reflects the organized thrombus and the heterogeneous T2‐FLAIR signal reflects the mixed composition of the sac. The lesion arises from the left middle cerebral artery and produces local mass effect on the adjacent parenchyma without surrounding vasogenic edema.

**FIGURE 2:**
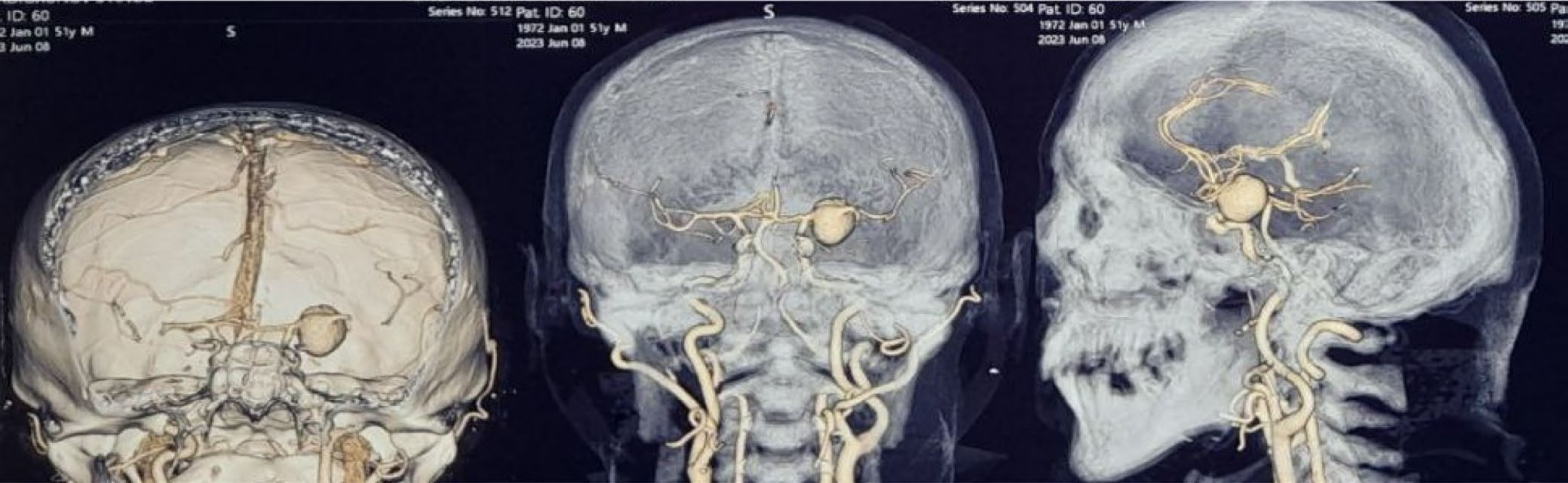
Cranial CT angiography of the cerebral vessels: Axial (first), coronal (middle), and sagittal (last) reconstructions demonstrating a giant left MCA aneurysm. The three orthogonal reconstructions display the fusiform morphology and the giant dimensions of the lesion, together with its continuity with the left middle cerebral artery. The contrast‐filled patent lumen is demarcated from the non‐enhancing mural thrombus, confirming the vascular nature of the lesion.

Subsequent brain magnetic resonance imaging (MRI) and multislice computed tomography (CT) angiography of the cerebral vessels demonstrated a giant fusiform aneurysm of the left middle cerebral artery (MCA) (Figures 1 and 2). MRI showed variable intraluminal signal characteristics, including T1 hyperintensity, T2 hypointensity, and heterogeneous T2‐FLAIR signal, suggesting thrombosis of most of the aneurysm sac. He was then referred to our center for further evaluation and treatment.

The differential diagnosis for a large, partially thrombosed lesion of this kind includes a thrombosed giant aneurysm, a hemorrhagic or necrotic neoplasm, and, less commonly, a cerebral abscess. The concentric, laminated intraluminal signal on MRI (T1 hyperintensity with T2 hypointensity), the absence of a solid enhancing tumor nodule or infiltrative perilesional edema, and the demonstration of a patent aneurysmal lumen in continuity with the MCA on CT angiography distinguished a partially thrombosed giant aneurysm from these mimics [14]. Correlation of the cross‐sectional imaging with the angioarchitecture of the parent vessel was therefore central to the diagnosis and to planning the staged reconstruction.

**FIGURE 3:**
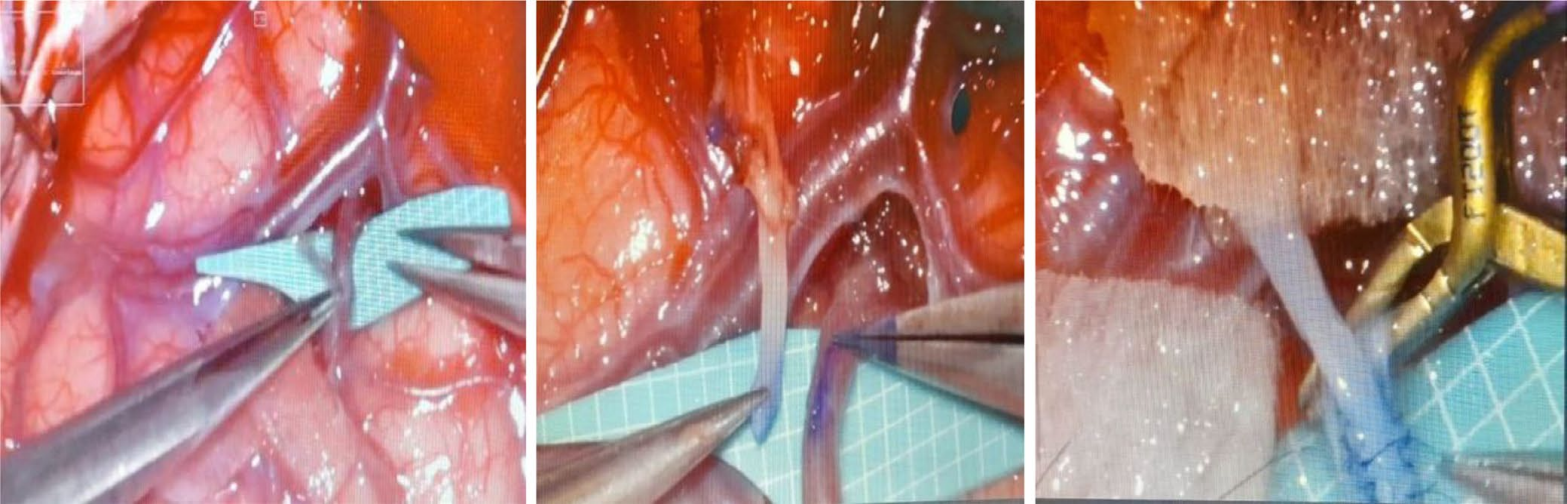
Intraoperative photos: (first) Isolation of the M4 segment of the MCA. (middle) Arteriotomy of the cortical artery. (last) Creation of an anastomosis. The frontal branch of the superficial temporal artery was prepared as the donor vessel and joined end‐to‐side to a cortical M4 recipient branch. The panels show, in sequence, dissection and isolation of the M4 recipient segment, the linear cortical arteriotomy, and the completed superficial temporal artery to M4 anastomosis, with the suture line along the anastomosis visible in the final panel.

Given the complex location of the aneurysm and the high procedural risk, a two‐stage treatment strategy was planned. In the first stage, the patient underwent left temporal craniotomy with creation of an extra‐intracranial microanastomosis. Specifically, an anastomosis was created between the frontal branch of the superficial temporal artery (STA) and the left M4 segment of the MCA (Figure 3). The patient tolerated the operation well and was extubated on the evening of postoperative Day 1. The postoperative course was uneventful, and his condition remained stable, although he experienced a moderate diffuse headache that resolved quickly. The bypass remained patent with preserved flow through the anastomosis. Patency of the anastomosis was confirmed intraoperatively by direct inspection of flow across the bypass under the operating microscope and postoperatively on MR angiography (Figure 6). The wound healed by primary intention without signs of inflammation, and the sutures were removed on postoperative Day 9. During the perioperative period, the patient received routine postoperative care with continuous neurological and hemodynamic monitoring; blood pressure was maintained in a normotensive range to preserve bypass patency while avoiding induced hypertension, and no vasospasm‐directed therapy was required given the absence of SAH.

**FIGURE 4:**
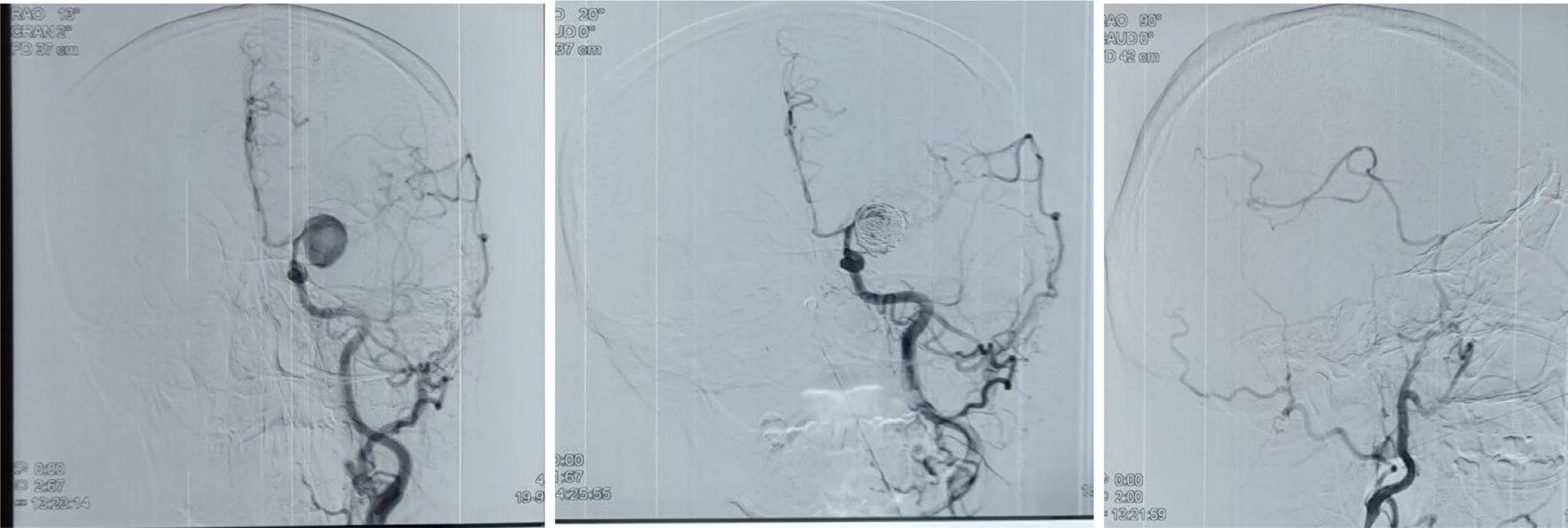
Selective carotid angiography before and after surgery. (first) Preoperative angiogram. (middle and last) Postoperative angiograms after endovascular exclusion of the left MCA fusiform aneurysm using detachable microcoils. The preoperative study demonstrates filling of the giant left middle cerebral artery aneurysm, while the postoperative studies, obtained after detachable microcoil embolization with occlusion of the M1 segment, show complete exclusion of the aneurysm from the circulation, corresponding to a Raymond–Roy Class I result.

**FIGURE 5:**
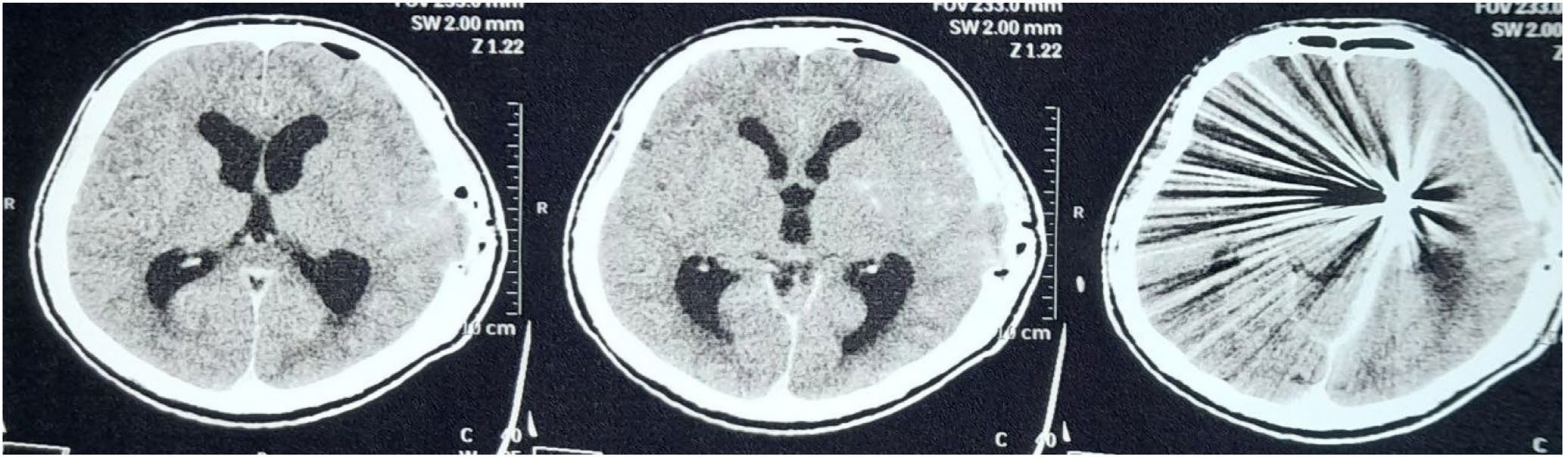
Multislice head CT axial sections, after endovascular exclusion of a fusiform aneurysm of the left middle cerebral artery using detachable microcoils. The high‐attenuation coil mass is seen within the treated aneurysm with associated beam‐hardening artifact. There is no new intraparenchymal hemorrhage and no established territorial infarction on these sections.

**FIGURE 6:**
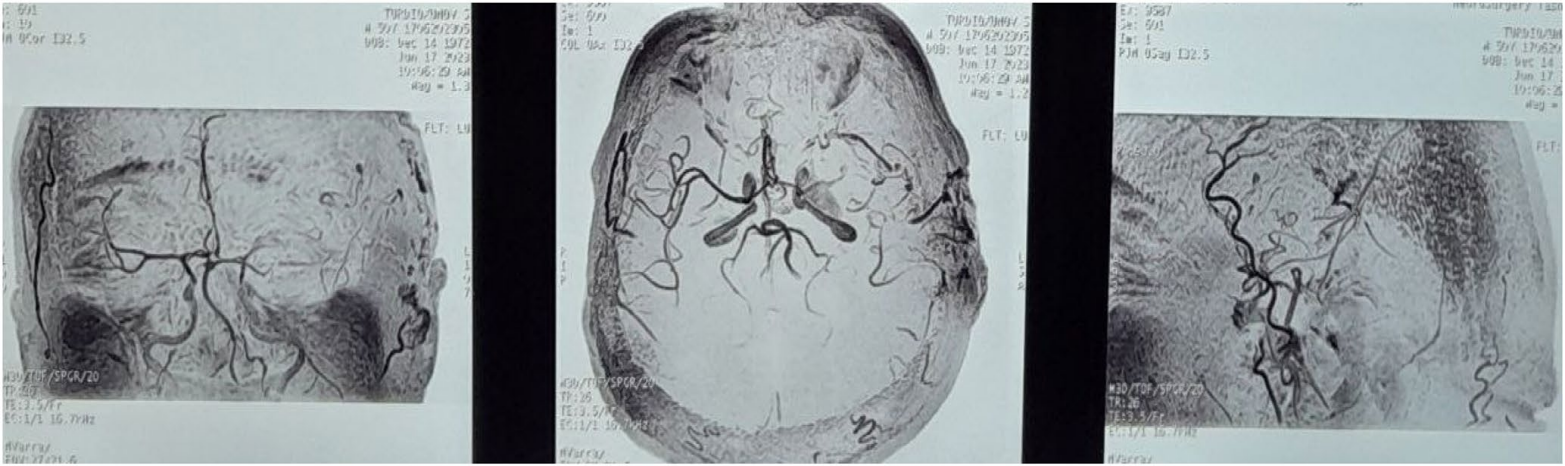
MR angiography and venography of the brain after left temporal craniotomy with creation of an extra‐intracranial microanastomosis. The magnetic resonance angiogram confirms patency of the superficial temporal artery–M4 bypass with preserved distal cortical flow and exclusion of the treated aneurysm, and the venographic sequences show patent dural venous sinuses without venous compromise.

The second stage was performed 3 days after the bypass. Endovascular treatment of the left MCA fusiform aneurysm was carried out with detachable microcoils, with intentional occlusion of the M1 segment of the parent MCA; the final angiogram demonstrated complete obliteration of the aneurysm, corresponding to Raymond–Roy Class I (Figure 4). Because the aneurysm was fusiform, the M1 segment was occluded together with the aneurysm rather than at a discrete neck. Large detachable coils were deployed within the aneurysm, and a small 3‐mm coil was placed in the distal M1 segment at its transition to the aneurysm, incorporating the parent M1 into the coil construct. A balloon test occlusion was not performed. Because the occlusion was limited to the distal M1 at the transition to the aneurysm, the lenticulostriate perforating arteries, which arise more proximally, remained patent. Postoperatively, the patient received supportive care. Follow‐up imaging included CT (Figure 5) and MRI angiography and venography (Figure 6).

## 4 Conclusion and Results (Outcome and Follow‐Up)

The postoperative period was uneventful. The patient was discharged in satisfactory condition, with neuroimaging findings unchanged from the immediate postoperative assessment. At 4‐month follow‐up, his condition remained stable, although he continued to report intermittent headaches.

### 4.1 Discussion

SAH due to rupture of cerebral aneurysms leads to significant disability and death even in surgically treated patients. Therefore, the choice of adequate treatment and understanding the factors associated with the development of intracranial aneurysms is of major clinical importance. In some patients, analysis of contributing factors does not support a dominant role for atherosclerosis or hypertensive vasculopathy, and key modifiable risks such as smoking or alcohol use may persist despite counseling. In such settings, an underlying genetic predisposition may warrant consideration. Given the potential for rupture and irreversible neurological sequelae, conservative management may be inappropriate in selected high‐risk patients, and proactive treatment strategies should be individualized based on aneurysm characteristics and overall rupture risk.

Currently, the main treatment principle for cerebrovascular aneurysms is aneurysm occlusion, which can be achieved using endovascular or microsurgical techniques [14]. However, after aneurysm management, complications can occur, including vasospasms. There is compelling evidence that cerebral vasospasm occurs due to SAH. Its management has evolved, and routine postoperative 3H therapy has largely been replaced by nimodipine, euvolemia, and targeted hemodynamic augmentation in selected patients.

Complex aneurysms are a rare cerebrovascular disease, and most have a poor prognosis. The development of giant aneurysms depends on their location, position relative to the subarachnoid space, the shape of the aneurysm (bulging, aneurysmal, or tortuous), and the presence of a number of anatomical features (thrombosis of the aneurysm, the presence of functionally important arteries emerging from the body of the aneurysm, etc.) [14].

According to the literature, the 5‐year mortality rate of patients with symptomatic giant aneurysms treated conservatively reaches 80%–100% when the aneurysm is in the vertebrobasilar territory. Surgical treatment of complex aneurysms usually involves open surgery to isolate the aneurysm from the blood supply, decompress the aneurysm sac, remove the thrombotic mass, and excise the aneurysm. Dome puncture is performed to reduce blood flow within the aneurysm, thereby reducing dome tension and facilitating microsurgical manipulations at the site of the parachoroidal aneurysm. The number of endovascular interventions for complex aneurysms is increasing, especially with the use of flow‐diverting stents. When complex aneurysm morphology is encountered, it is important to remember that microsurgical and endovascular strategies can be combined, and revascularization surgery should be considered in particular.

The rationale for the staged strategy adopted here, low‐flow STA to M4 revascularization followed by endovascular coil exclusion, was driven by the morphology of the lesion and by the anatomy of the MCA bifurcation. A large organized thrombus burden limits the durability of purely reconstructive endovascular options: in a multicenter cohort of partially thrombosed aneurysms treated with flow diversion, pretreatment thrombosis exceeding 50% of the aneurysm was associated with a significantly lower rate of complete occlusion (58.8% vs. 87.1%), and ischemic complications occurred in nearly 1 in 10 patients [16]. Flow‐diverter reconstruction is further complicated at bifurcation aneurysms, where efferent branches and perforators covered by the device are placed at risk and incomplete obliteration may predispose to delayed growth or rupture. Establishing distal flow before the aneurysm is addressed converts an otherwise high‐risk deconstructive maneuver into a controlled one, because the M4 territory is protected against ischemia should prolonged parent‐vessel occlusion or sacrifice become necessary [14, 17]. This insurance‐and‐replacement principle, in which a low‐flow STA‐MCA bypass secures the distal territory and the aneurysm is then excluded by microsurgical or endovascular means, has been applied successfully to complex anterior‐circulation aneurysms, including hybrid series that combine STA‐MCA bypass with endovascular embolization of the aneurysm and parent artery [17, 18, 19]. In the present patient, in whom the M1 segment of the parent artery was intentionally occluded during the endovascular stage, the low‐flow bypass provided flow replacement to the distal territory with lower operative morbidity than a high‐flow interposition graft, and staging the definitive occlusion endovascularly avoided direct microsurgical manipulation of a thrombosed, broad‐based dome.

Durable surveillance of both the bypass and the treated aneurysm is essential, because more than 20% of endovascularly treated aneurysms recur and partially thrombosed lesions are particularly prone to recanalization and continued growth [16, 20]. Digital subtraction angiography remains the reference standard for confirming graft patency and for detecting residual or recurrent aneurysm filling, whereas noninvasive modalities are constrained in this setting: the platinum coil mass generates beam‐hardening artifact on CT angiography (evident on the postoperative study, Figure 5) and a susceptibility signal void on time‐of‐flight MR angiography that can obscure a small neck remnant [20]. A pragmatic scheme therefore pairs an early catheter angiogram to establish the postoperative baseline with subsequent noninvasive follow‐up, reserving repeat DSA for any suspected recurrence. Given the persistence of headache at 4 months, continued clinical and imaging follow‐up is warranted to exclude aneurysm regrowth or bypass compromise as a contributing cause.

### 4.2 Limitations

This report has several limitations. It describes the outcome of a single patient, which precludes generalization and does not permit any comparative assessment of the staged strategy against alternative reconstructive or endovascular approaches. Follow‐up was limited to 4 months, an interval too short to establish the durability of aneurysm exclusion or the long‐term patency of the bypass, and the headache reported at last contact remains incompletely explained. Finally, the metallic artifact generated by the coil mass constrains noninvasive imaging surveillance, so that confident exclusion of a residual neck or early recurrence may require catheter angiography. In addition, objective intraoperative flow quantification of the bypass (e.g., indocyanine green videoangiography or quantitative flow measurement) was not performed.

### 4.3 Conclusions

The question of the optimal treatment of cerebral aneurysms remains complex and must be individualized according to aneurysm morphology, vascular anatomy, and patient‐specific risk factors. The presented clinical case demonstrates the feasibility of staged microsurgical and endovascular treatment of a giant complex left MCA aneurysm using craniotomy with EC‐IC microanastomosis followed by endovascular exclusion. This treatment strategy was technically demanding but allowed preservation of distal cerebral perfusion and definitive exclusion of the aneurysm. In selected patients with complex cerebrovascular pathologies, revascularization can expand surgical options and improve treatment safety when conventional techniques alone may be insufficient.

## Author Contributions

**Ruzikulov M. Mazhidovich:** conceptualization, investigation, writing – original draft. **Kashif Qureshi:** supervision. **Kariev G. Maratovich:** software, formal analysis. **Rasulov S. Orzukulovich:** formal analysis, data curation. **Matmusaev M. Makhmudovich:** data curation, methodology. **Murtaja Satea:** visualization. **Khazratkulov R. Bafoevich:** data curation, methodology. **Matmusaev M. Makhsudovich:** visualization. **Ismail Bozkurt:** supervision. **Kivanc Yangi:** supervision. **Bipin Chaurasia:** supervision, visualization, validation, writing – review and editing.

## Funding

The authors have nothing to report.

## Consent

Written informed consent was taken from patient prior to initiation of this project. Written informed consent from the patient was obtained for publication according to journal guidelines.

## Data Availability

Data sharing is not applicable to this article as no datasets were generated or analyzed during the current study.
